# Gamma knife radiosurgery (GKRS) for pineal region tumors: a study of 147 cases

**DOI:** 10.1186/s12957-015-0720-5

**Published:** 2015-10-21

**Authors:** Wentao Li, Binfei Zhang, Wenxing Kang, Boning Dong, Xudong Ma, Jinning Song, Yonghong Liu, Zhenqiang Liang

**Affiliations:** Department of Neurosurgery, the First Affiliated Hospital, Medical School of Xi’an Jiaotong University, #277, Yantaxi Rd, Xi’an, 710061 China; Honghui Hospital, Xi’an Jiaotong University Health Science Center, Xi’an, 710054 China; Department of Radiation Oncology, 323 Hospital of Chinese People’s Liberation Army, Xi’an, 710000 China; Beilin Kangjie Hospital, Xi’an, 710000 China; Department of Neurosurgery, the Northwest Civil Aviation Hospital, Xi’an, 710061 China; Department of Neurosurgery, the Dingxi First People’s Hospital, Dingxi, 743000 China

**Keywords:** Gamma knife radiosurgery, Pineal region tumors, Germ cell tumors, Follow-up

## Abstract

**Background:**

The purpose of this study is to evaluate the effectiveness of gamma knife radiosurgery (GKRS) in the treatment of pineal region tumors (PRTs).

**Methods:**

We retrospectively reviewed 147 cases of PRTs primarily treated with GKRS at our hospital between 1999 and 2009. Mean follow-up time was 67 months (range 60.5–100.1). The local tumor control rates (LTCRs) and overall survival rates were calculated to evaluate the results of the GKRS treatment.

**Results:**

At 2 months after GKRS, tumor volume was significantly reduced in 91 cases (61.9 %). At 6 months, average tumor volume was 4.2 cm^3^ as compared to 8.47 cm^3^ before GKRS. By 1 year after GKRS, the tumor completely disappeared in 57 patients. Fourteen patients underwent second treatment, and one patient had third treatment. The overall survival rates were 72.1 % at 3 years and 66.7 % at 5 years for all patients and 62.4 % at 3 years and 54.5 % at 5 years for germ cell tumors (GCTs). The LTCRs were 94.30 % at 3 years and 90.80 % at 5 years for all patients and 88.00 % at 3 years and 77.27 % at 5 years for GCTs.

**Conclusions:**

GKRS is an effective and safe modality that can be widely used to PRTs as the primary therapy.

## Background

The pineal region tumors (PRTs) are a deep-seated, heterogeneous group of mass lesions, which are derived from cells located in or adjacent to the pineal gland. PRTs are rare, which accounts for 0.5–1 % of all intracranial tumors in the United States and Europe [1] but have a higher incidence of 3.2 % in Japan [2, 3], 0.9 % in Islamabad [4], 1.2 % in Karachi [5], and 1.9 % in China [6]. These tumors are approximately ten times more common in children than in adults and constitute 3–8 % of pediatric intracranial tumors [7].

Due to heterogeneous cellular origins, PRTs represent a spectrum of neoplasms ranging from benign (teratoma, meningioma, pineocytoma, ependymoma, etc.) to malignant (pineoblastoma, malignant teratoma, malignant germ cell tumors, etc.). The management strategies for these tumors remain controversial [8]. Some investigators state that surgical resection should be used as the initial therapeutic modality [9–11] and that histologic diagnosis of PRTs is required for rational treatment. In contrast, some Asian investigators [12] recommend a test dose of radiation therapy based on the high incidence of germinoma in their countries. There are regional differences in the incidence of GCTs in the pineal region. In Asian, germ cell tumors (GCTs) are more common overall (accounting for 3/1 % of all primary brain tumors in Japan [13]/China, respectively [6]) but are predominantly the more benign germinomas. In Japan, GCTs account for 70.3 % of pineal region tumors [6, 13]. Some experts in Japan advocate empiric radiosurgery [14, 15] for reducing the cost of treatment and the need for hospitalization. Several clinical studies have demonstrated that gamma knife radiosurgery (GKRS) has high efficacy and low morbidity [16], and thus has the potential as a primary treatment modality [17, 18].

On the whole, GKRS is safe and effective for pineal region tumors. The most successful GKRS treatment of pineal region tumors has been with benign or low-grade lesions. The three broad categories of tumor treated by GKRS include pineal parenchymal tumors (pineocytomas and pineoblastomas), GCTs (germinomatous and nongerminomatous), and glial tumors (astrocytomas).Pure germinomas are exquisitely radiosensitive. Many studies [11, 18–20] mainly used GKRS as an adjuvant therapy rather than as a primary treatment modality. Only a few studies explore the efficacy of GKRS in treating pineal region tumors in series as a primary method, and the numbers of cases were small [19–21]. There is a need for larger studies with longer follow-up before GKRS becomes widely adopted in the treatment of pineal region tumors. In this report, we reviewed our institutional experiences of treating pineal region tumors by GKRS in 147 patients.

## Methods

### Ethics, consent, and permissions

This study was approved by the First Affiliated Hospital of Xi' an Jiaotong University Institutional Review Board and met the requirements of medical ethics. Consents to participate the study from the participants (or legal parent or guardian for children) were obtained.

### Consent to publish

We had obtained consent to publish from the participants (or legal parent or guardian for children) to report individual patients’ data in any form (including images, videos, voice recordings, etc.).

### Patient population and diagnosis

We retrospectively reviewed 147 consecutive cases of pineal region tumors treated with GKRS at the First Affiliated Hospital of Xi’an Jiaotong University (Xi’an, China) between 1999 and 2009. All subjects were Han Chinese from Northwest China. Inclusion criterion is: all patients were newly diagnosed incident PRT cases undergoing GKRS at our institution. Exclusion criterion: patients with arteriovenous malformations and brain metastases.

Diagnosis was mainly based on magnetic resonance imaging (MRI; GE HDxt 3.0T, USA) combined with medical history, clinical presentations, Karnofsky performance score [22], and patient age. T1-weighted and T2-weighted MRI were performed. MRI scans were assessed independently by two radiologists blinded to the clinical outcome.

### GKRS technique

Based on data from computer tomography (CT) and MRI, patients of progressive hydrocephalus underwent ventricular peritoneal (VP) shunt 7 days before GKRS (Elekta Instruments, Atlanta, GA, USA). The Leksell GammaPlan treatment planning software (versions 4.10–5.34, Elekta, Sweden) was used on all patients. Between 1 and 9 isocenters (mean 6.24 isocenters) were used in treatment planning. The Automated Positioning System (Elekta) was used to streamline patient positioning. Central dose was 25–40 Gy (mean ± SD 36.34 ± 2.11 Gy), and marginal dose was 9–15 Gy (mean ± SD 13.6 ± 1.02 Gy).

### Patient assessment and follow-up

The duration of follow-up ranged from 60.5 to 100.1 months (mean 67 months). The results of GKRS were mainly evaluated by changes in tumor size on MRI at 1, 3, 6, and 12 months after GKRS. Meanwhile, the neurological presentations were evaluated. The systemic criteria proposed by the Japan Brain Tumor Registry [23] were used. This system is composed of five grades including complete response (CR; tumor disappearance), partial response (PR; ≥50 % tumor volume reduction), minor response (MR; 25–50 % reduction), no change (NC; <25 % reduction), and progression (tumor enlargement). The local tumor control rate was calculated as (CR + PR + MR + NC)/total.

## Results

### Patients’ characteristics

We identified 158 lesions in the 147 patients included in this study. Eight patients had multiple lesions. Most lesions were located in the pineal region. The demographics/clinical presentation of patients are summarized in Table 1. There were 105 males and 42 females, with a ratio of 2.5:1. Patients’ ages ranged from 6 to 74 years (mean ± SD 35.6 ± 12.1 years). The mean volume of PRTs before GKRS treatment was 8.47 cm^3^ (SD 6.22 cm^3^). The duration between the onset of symptoms and admission at our institute ranged from 3 months to 2 years. The most common symptoms were headache, nausea, and vomiting. Other symptoms included polydipsia, polyuria, ataxia, eye movement disorders, parinaud syndromes, precocious puberty, and hypogenesis.

**Table 1:** Clinical presentations of patients with germ cell tumors (GCTs) and non-germ cell tumors (non-GCTs)

Classification	Non-GCTs	GCTs
No. of cases	107	40
Age	42.3 ± 10.2	14.4 ± 6.1
Minimum age	17	6
Maximum age	74	20
Sex		
Male	74	31
Female	33	9
Clinical symptoms		
Headache	59	38
Nausea and vomiting	40	29
Polydipsia and polyuria	9	32
Eye movement disorders	23	18
Parinaud sindromas	16	19
Precocious puberty	2	1
Hypogenesis	1	4
Ataxia	15	12

### GKRS treatment

Patients first underwent MRI assessment to locate brain tumors. The average Karnofsky score was 72.23. A total of 104 (66 %) lesions occurred in adults and 54 (34 %) occurred in children. The lesions showed clear boundary. Some lesions had cystic changes, and gadodiamide-enhanced (OmniScan, GE Healthcare) MRI showed uniform enhance mentor partial enhancement in cystic areas. Eight patients had multiple lesions located in the pineal region, parietal lobe, and occipital lobe. Forty patients were diagnosed as germ cell tumors. The mean tumor volume was 8.47 cm^3^ (0.1–29.2 cm^3^), and the maximum tumor diameter was 3.8 cm. Dosimetry of GKRS is listed in Table 2.

**Table 2:** Dosimetry of GKRS

Items	Mean ± SD
Tumor volume (cm^3^)	8.47 ± 6.22
No. of isocenters	6.24 ± 1.11
Central dose (Gy)	36.34 ± 2.11
Marginal dose (Gy)	13.6 ± 1.02
Maximum dose (Gy)	28.35 ± 5.37

*GKRS* Gamma knife radiosurgery, *SD* standard deviation

### Follow-up

After GKRS treatment, three patients developed complications including severe headache, projectile vomiting and subsequent coma and were transferred to the intensive care unit. These patients were mainly given life-sustaining treatment and treatment to reduce intracranial pressure. One patient developed acute brain herniation due to rapidly progressing severe brain edema and died. The other two patients regained consciousness after their brain edema disappeared and were transferred to a general ward. They did not experience any other discomfort during the follow-up period after leaving the hospital. Fatigue was seen in 103 patients, headache in 97 patients, and nausea and vomiting in 69 patients. After reducing intracranial pressure using mannitol, these symptoms were alleviated and gradually disappeared.

At 2 months after GKRS, lesions in 91 cases (61.9 %) were significantly reduced, and symptoms were improved or disappeared in 121 cases (83.31 %). At 6 months, radiographic review showed that the average tumor volume was reduced to 4.2 cm^3^ (range 0–19.7 cm^3^) from 8.47 cm^3^ before GKRS. The pineal region tumor volume was reduced, and saddle or occipital lesions disappeared in 123 cases (83.67 %). Tumors in 17 cases increased in size, but tumor density was reduced in 14 of these cases. The average follow-up duration was 67.2 months (range 60.5–100.1). The tumor disappeared in 57 patients after 1 year (Table 3). The overall survival rates for 1, 3, and 5 years after GKRS were 80.2, 72.1, and 66.7 %, respectively. Local tumor control rates were 97.40, 94.30, and 90.80 % for 1, 3, and 5 years, respectively.

**Table 3:** Evaluation of all (147) cases after GKRS treatment

	CR (%)	PR (%)	MR (%)	NC (%)	PROGR (%)	Total	LTCR (%)	ATV (cm^3^)	Karnofsky scoring	Survival rate (%)
Before treatment								8.47	72.23	
1 year after treatment	57 (48.31)	34 (28.81)	22 (18.64)	2 (1.69)	3 (2.54)	118	97.40	1.23	87.83 ± 7.31	80.2
3 years after treatment	52 (49.06)	27 (25.41)	19 (17.92)	2 (1.89)	6 (5.66)	106	94.30	1.33	77.25 ± 13.38	72.1
5 years after treatment	43 (43.88)	26 (26.53)	17 (17.35)	3 (3.06)	9 (9.18)	98	90.80	2.29	76.43 ± 17.76	66.7

*Abbreviations: GKRS* Gamma knife radiosurgery, *CR* complete response (tumor disappearance), *PR* partial response (≥50 % tumor volume reduction), *MR* minor response (25–50 % reduction), *NC* no change (<25 % reduction), *PROGR* progression (tumor enlargement), *LTCR* local tumor control rate = CR + PR + MR + NC/total, *ATV* average tumor volume

We also analyzed the GCT cases separately. In the GCT sub-group (40 cases), symptoms were improved in 13 cases and completely disappeared in 24 cases at 2 months post GKRS. At 6 months, lesions disappeared in eight cases according to MRI scans. Another 8 cases showed residual lesions and underwent second radiosurgery. There was no death for the 40 cases of GCTs. The 1-, 3-, and 5-year overall survival rates for GCTs (Table 4) were 77.5, 62.4, and 54.5 %, respectively. The local tumor control rates were 96.70, 88.00, and 77.27 %, for 1, 3, and 5 years, respectively.

**Table 4:** Evaluation of GCTs (40 cases) after GKRS treatment

	CR (%)	PR (%)	MR (%)	NC (%)	PROGR (%)	Total number	LTCR (%)	ATV (cm^3^)	Karnofsky scoring	Survival rate (%)
Before treatment								7.28	76.25	
1 year after treatment	14 (45.16)	12 (38.71)	2 (6.45)	2 (6.45)	1 (3.23)	31	96.70	2.21	85.22 ± 9.15	77.5
3 years after treatment	11 (44.00)	8 (32.00)	1 (4.00)	2 (8.00)	3 (12.00)	25	88.00	2.54	72.52 ± 14.58	62.4
5 years after treatment	8 (36.36)	7 (31.82)	1 (4.55)	1 (4.55)	5 (22.73)	22	77.27	2.91	66.14 ± 16.86	54.5

*Abbreviations: GCTs* germ cell tumors, *GKRS* gamma knife radiosurgery, *CR* complete response (tumor disappearance), *PR* partial response (≥50 % tumor volume reduction), *MR* minor response (25–50 % reduction), *NC* no change (<25 % reduction), *PROGR* progression (tumor enlargement), *LTCR* local tumor control rate = CR + PR + MR + NC/total, *ATV* average tumor volume

Fourteen patients in this study (eight of GCTs and six of non-GCTs) underwent second treatment, and one of these patients had third treatment. Eight of these patients had multiple lesions that are either GCTs or had a total volume of 21.5–28.1 cm^3^. One patient had polyuria during the first 3 months after treatment. The patients’ MRI revealed a new lesion in suprasellar region oppressing pituitary stalk. Since the volume of pineal region lesion did not reduce, this patient was given low dose of whole brain radiation therapy. Five months later, polyuria and the lesion in suprasellar region disappeared, and pineal region tumor shrank.

## Discussion

In this study, we reviewed the results of GKRS in 147 patients. The average duration of follow-up was 67.2 months. The 3/5-year survival rates for patients after GKRS treatment were 72.1/66.7 % for all patients and 62.4/54.5 % for GCTs. The 3/5-year local tumor control rates were 94.30/90.80 % for all patients and 88.00/77.27 % for GCTs, respectively.

The results indicate that GKRS without histological verification of PRTs is acceptable. It has been controversial whether histological diagnosis before treatment is necessary [10, 18, 24] in the management of PRTs, partly owing to racial differences in incidence [1–3, 6]. Therefore, it is not clear which treatment options are most beneficial.

With the advances of clinical image (MRI) and clinical features, we can now diagnose PRTs and differentiate GCTs from non-GCTs without histological analysis. Therefore, GKRS can be performed either as a primary therapy or as an adjuvant therapy for conventional treatments. Kanamori [24] reported that the histological diagnoses of 38 out of 39 GCT patients and all of eight non-GCT patients were similar to their clinical diagnoses. Konovalov reported that [10] surgery or GKRS for patients with butterfly-shaped radiologic sign in MRI were successful without pathologic confirmation of diagnosis. Our institutional experiences support this option.

In our study, the 5-year survival rate for patients with PRTs is 66.7 %; however, the survival rate in Kanamori’s study [24] was 94 % without histological verification. The difference in survival rate could be due in part to the fact that there was no neoadjuvant chemotherapy in our study. Alternatively, it could be due to differences in the number of cases (147 cases in our study and 39 cases in Kanamori’s) or patient population. The 5-year local tumor control rate in our study was 90.80 %, which is higher than 70 % in an earlier study of 49 cases [20]. In the present study, the 1-, 3-, and 5-year overall survival rates were 77.5, 62.4, and 54.5 %, respectively, in GCTs cases (40 cases) after GKRS. Mori reported that [20] survival rates at 5 and 10 years after GKRS in GCT cases (*n* = 38) were both 68 %. The difference in survival rate could be due in part to diagnosis. In 13 of 38 patients was histologically diagnosed, but in others GCT was diagnosed by elevation of serum tumor markers or clinical course. In our study, GCT was diagnosed by MRI, medical history, and clinical presentations.

A definitive histological verification is surely desirable, but successful treatment methods without the need for biopsy have been developed [24, 25]. It is interesting that the survival rates are not significantly influenced by the unclear histological diagnosis. This phenomenon likely results from the large variety of tumor types in the pineal region running necessary biopsies in some circumstances.

With the development of neurosurgery and the emergence of CT, MRI, and surgical microscope, it is possible to achieve complete removal and secondary complete removal. Therefore, there has been a significant decrease in death rate. Microsurgical mortality is under 8 % and disability rate is under 12 % [26]. Radiotherapy is safe and effective, which makes it a good alternative treatment, especially for germ cell tumors that are sensitive to radiation. There are still disagreements over the treatment of pinealoma. Considering the efficacy, imbalance of domestic economic development, as well as cost-effectiveness of surgical total resection and subtotal resection in developing countries [27], it is difficult to achieve a wide range of application for surgical total resection and subtotal resection. As such, radiosurgery cannot be replaced by surgery because it is a non-time-consuming, economic, and less invasive therapy.

GKRS can not only kill the tumor cells but also reduce the damage to the surrounding normal brain tissues [28], thanks to its technical characteristic of large dose in targeted area and low dose on the edge [29]. Therefore, GKRS is associated with less systemic side effects compared to conventional radiation. Moreover, it can also be used to treat tumors that are insensitive to conventional radiotherapy.

Pinealoma is usually accompanied by obstructive hydrocephalus. Sixteen patients (10.9 %) in this study had Parinaud’s syndrome, which is a sign of acute obstructive hydrocephalus. In order to prevent perioperative and postoperative intracranial hypertension, VP shunt was performed on patients with progressive hydrocephalus. In the present study, patients treated with mannitol after surgery had no intracranial hypertension, infection, or shunt malfunction. These findings indicate that we can improve the safety of GKRS by actively dealing with hydrocephalus.

Given that pinealoma may metastasize by seeding through the cerebrospinal fluid pathways, there is a debate about whether radiotherapy or chemotherapy should be used. The rate of metastases from pinealoma is continually rising based on recent reports. Ogawa et al. [30] reviewed 126 cases of intracranial germ cell tumors that underwent full central radiotherapy and found that the incidence of spinal relapses was 4 % (2 of 56) for patients treated with spinal irradiation and 3 % (2 of 70) for those without spinal irradiation. Nguyen et al. [31] reviewed 21 patients with intracranial germinoma treated with radiotherapy and found that irradiation with chemotherapy is associated with increased rates of failures in the brain and spine.

## Conclusion

In conclusion, our institutional experiences support that GCTs may be diagnosed based on MRI and clinical presentations without biopsy or surgery. Moreover, GKRS appears to be an effective, low-risk treatment option that can be widely used for pineal region tumors either as a primary therapy without histological diagnosis. Further studies are needed to evaluate the effectiveness of GKRS for the treatment of PRTs as a primary therapy in more details.

## Abbreviations

CR: Complete response
CT: Computer tomography
GCTs: Germ cell tumors
GKRS: Gamma knife radiosurgery
LTCRs: Local tumor control rates
MR: Minor response
NC: No change
PR: Partial response
PRTs: Pineal region tumors
VP: Ventricular peritoneal
